# Editorial: Do Both Psychopathology and Creativity Result from a Labile Wake-Sleep-Dream Cycle?

**DOI:** 10.3389/fpsyg.2017.01824

**Published:** 2017-10-20

**Authors:** Sue Llewellyn, Martin Desseilles

**Affiliations:** ^1^Humanities, University of Manchester, Manchester, United Kingdom; ^2^Psychology, University of Namur, Namur, Belgium

**Keywords:** psychopathology, creativity, state de-differentiation, labile sleep, creative insight

We invited contributions on the following hypothesis: the boundaries between wake, sleep, and dreaming (W/S/D) can become less distinct, engendering a de-differentiated, disordered state in which wake, sleep and dreaming become interpenetrated (see Llewellyn, 2016, for a review). In other words, the state differentiation which, through differing neuromodulation and input-output gating, achieves the W/S/D cycle is disrupted. Consequently, the mind/brain assumes more of a hybrid, de-differentiated wake-sleep-dream state across the cycle. Due to the chaos dynamics of brain processes, where a small change can engender diverse results, de-differentiation can take a variety of forms. A slight to moderate degree of penetration of wake by REM sleep may result in enhanced creativity. Normal waking consciousness is a sub-critical, ordered state (Carhart-Harris et al., 2014) whereas creative states are critical. Creative insight depends on spreading neural activation to make remote associations amongst memory (including knowledge) elements (Yaniv and Meyer, 1987; Dijksterhuis and Meurs, 2006; Dijksterhuis and Nordgren, 2006; Baird et al., 2012; Ritter and Dijksterhuis, 2014). Hyperassociativity, or the ability to make remote associations, is more characteristic of rapid eye movement (REM) sleep and dreaming than either wake or non-REM sleep (Stickgold et al., 1999; Walker et al., 2002; Cai et al., 2009; Sterpenich et al., 2014). Therefore, if REM sleep begins to suffuse wake, enhanced creativity would be anticipated. But moderate to severe interpenetration of W/S/D may precipitate psychopathologies- as the distinct functions of W/S/D are partially eroded. In ADHD, for example, the W/S/D cycle is de-differentiated or labile (Kirov and Brand, 2014). If our proposal is correct an association between creativity and psychopathology would be observed. Evidence for this is accumulating, see for example, Kyaga et al. (2011), Santosa et al. (2007), and Rybakowski and Klonowska (2010).

Our contributors focus on different aspects of the complex interrelationships outlined above. First, to explicate the relationship between labile sleep and creative insight, Kirov et al. operationalized a labile sleep cycle as the rate of sleep stage transitions. They found a strong positive correlation between the rate of NREM/REM transitions and insight, after sleep, into a hidden sequence in a serial reaction time task. Second, both creative insights and psychopathologies have long thought to depend on access to, normally, unconscious associations (Ghiselin, 1952; Maher, 1972). De-differentiation of W/S/D would enable conscious access in wake to the remote associations made during REM sleep. Whereas, in a normal, differentiated sleep cycle, these associations, if retained, would remain unconscious. Yordanova et al. provide a detailed understanding of the interactions between conscious and unconscious knowledge, before, during and after sleep, which underlie insight performance on a visual serial reaction time task. Third, dreams have long been thought to embed personal insights. Bob and Louchakova argue for common brain processes in dreaming and dissociative states, noting that creativity during dreaming may take the form of personal insights as experiential memories which are only remotely associated become integrated in dream imagery. Continuing on the personal insight theme, Edwards et al. compare the personal insight gains when participants consider a recent dream as compared to a recent event. Using the Ullman method, self-assessment of personal insight after considering dream content was significantly higher than after considering a real life event.

Fourth, if dream and wake states become hybridized, psychopathologies may arise. Skrzypińska and Szmigielska argue those with Borderline Personality Disorder experience dream-reality confusion- defined as difficulty determining whether an event actually occurred or was an aspect of a dream. Some of those with psychopathologies are also creative, van Heugten - van der Kloet et al. explored the relationship between sleep, creativity and dissociation. They found a relationship between acute dissociative symptoms and creativity. McNamara and Bulkeley focus on the creativity of dreams and the psychopathology of religious delusions. They contend that supernatural agent cognitions link the two when REM sleep begins to pervade waking consciousness. Continuing to interrogate REM sleep, Hutchison and Rathore are interested in REM theta, noting that theta oscillations are also prominent during exploration during wake. During REM sleep, they argue that theta may drive novel associations amongst emotionally salient memory elements. Such a process may be sleep-dependent, mental exploration equating to the spreading neural activation which characterizes creative insight. Fifth and finally, two papers provide a detailed understanding of hyperassociativity during dreaming. Horton and Malinowski focus on novel associations and re-combinations of personal memory elements in dreams, arguing such re-combinations constitute creative insights which are more useful in future contexts than whole, veridical memories. Malinowski and Horton explore the links between identifying novel associations during dreams and metaphorical thinking, noting that a metaphor provides insight into how two or more entities are associated. They argue that dream metaphors derive from novel associations and can generate new, creative ideas.

Overall, the most prominent theme in our collection is that of creative or personal insight. The distinction between these two may be one of emphasis and extension. Individuals can have creative insights which extend, in the future, to socially useful innovations. But such insights depend, originally, on personally held knowledge. With an emphasis on knowledge about the person, insights can give rise to new personal knowledge which can be mobilized in a therapeutic context. Several of our papers lend support to the idea that REM sleep and dreaming engender creative insights. With a labile, de-differentiated W/S/D cycle such insights would become additionally available in wake. Our collection also extends the emergent literature on sleep, creativity and psychopathology through linking the three. However, only one paper, Bob and Louchakova, touched upon the chaos dynamics which may underlie de-differentiation. To progress work on the possible links between creativity, psychopathologies and the de-differentiation of W/S/D, interdisciplinary research on labile sleep, chaos dynamics and REM hyperassociativity is called for.

## Author contributions

Both authors contributed to writing up the topic, handling the reviews and writing up the editorial.

### Conflict of interest statement

The authors declare that the research was conducted in the absence of any commercial or financial relationships that could be construed as a potential conflict of interest.
